# Reproducibility and Concordance of Differential DNA Methylation and Gene Expression in Cancer

**DOI:** 10.1371/journal.pone.0029686

**Published:** 2012-01-03

**Authors:** Chen Yao, Hongdong Li, Xiaopei Shen, Zheng He, Lang He, Zheng Guo

**Affiliations:** 1 Bioinformatics Centre and Key Laboratory for NeuroInfomation of the Education Ministry of China, School of Life Science, University of Electronic Science and Technology of China, Chengdu, China; 2 Colleges of Bioinformatics Science and Technology, Harbin Medical University, Harbin, China; King Faisal Specialist Hospital & Research Center, Saudi Arabia

## Abstract

**Background:**

Hundreds of genes with differential DNA methylation of promoters have been identified for various cancers. However, the reproducibility of differential DNA methylation discoveries for cancer and the relationship between DNA methylation and aberrant gene expression have not been systematically analysed.

**Methodology/Principal Findings:**

Using array data for seven types of cancers, we first evaluated the effects of experimental batches on differential DNA methylation detection. Second, we compared the directions of DNA methylation changes detected from different datasets for the same cancer. Third, we evaluated the concordance between methylation and gene expression changes. Finally, we compared DNA methylation changes in different cancers. For a given cancer, the directions of methylation and expression changes detected from different datasets, excluding potential batch effects, were highly consistent. In different cancers, DNA hypermethylation was highly inversely correlated with the down-regulation of gene expression, whereas hypomethylation was only weakly correlated with the up-regulation of genes. Finally, we found that genes commonly hypomethylated in different cancers primarily performed functions associated with chronic inflammation, such as ‘keratinization’, ‘chemotaxis’ and ‘immune response’.

**Conclusions:**

Batch effects could greatly affect the discovery of DNA methylation biomarkers. For a particular cancer, both differential DNA methylation and gene expression can be reproducibly detected from different studies with no batch effects. While DNA hypermethylation is significantly linked to gene down-regulation, hypomethylation is only weakly correlated with gene up-regulation and is likely to be linked to chronic inflammation.

## Introduction

Methylation arrays have been used to identify hundreds of genes with differential DNA methylation of their promoters in various types of cancers [1], [2], [3], [4], hereafter referred to as DM genes, providing insights into cancer biology and useful biomarkers for predicting cancer outcomes and drug targets [5]. However, various biological and technical factors may affect the discovery of biomarkers for human cancers. In particular, batch effects, which could be introduced by using samples from different experimental batches (such as sample preparation at different times, with different protocols, on different chip lots or different microarray platforms), may produce systematic non-biological differences between different groups of samples [6], [7], [8], [9]. Thus, a challenging task of fundamental importance for biomarker validation is to evaluate the reproducibility of DM gene discovery across different studies for a particular cancer [10], [11], [12]. This problem has not been fully addressed until now. Once DM genes are reproducibly identified for a particular cancer, an important task is to define their roles in cancer development. It is widely accepted that aberrant promoter methylation is a significant cause of altered gene expression in cancer [13]. However, several recent studies have challenged the inverse correlation between methylation and expression changes [14], [15]. Thus, the relationship between changes in DNA methylation and gene expression in cancer still needs to be systematically evaluated.

The Cancer Genome Atlas (TCGA) database [16] provides hundreds of methylation profiles for various cancer types. For a particular cancer, samples were often collected from different laboratories and treated in different experimental batches due to practical complications such as technology limitation. In this paper, based on methylation profiles for nine types of cancer collected in the TCGA database [16], we showed that improperly integrating data from different experimental batches to extract DM genes could be misleading. After excluding datasets with potential batch effects, we demonstrated that the change of methylation states (hypermethylation or hypomethylation) of DM genes in cancer samples compared with normal samples can be highly reproducibly detected from different datasets for a given cancer. A similar trend was observed for the expression changes of genes in cancers based on datasets available from the Gene Expression Omnibus database [17]. Then, based on the reproducible DM and DE genes for each cancer type, we determined that the promoter hypermethylation is highly inversely correlated with the gene down-expression, whereas hypomethylation is only weakly correlated with the up-expression of genes with large expression changes. At last, we found that hypomethylated genes mainly disturb functions directly linked to chronic inflammation, such ‘chemotaxis’ and ‘immune response’ functions.

## Materials and Methods

### Data sources

The expression and methylation datasets described in Table 1 and Table 2 were downloaded from the GEO [17] and TCGA [16] databases, respectively. The raw gene expression profiles were normalised using the robust multi-array analysis (RMA) algorithm [18]. We used the level 2 data defined in the TCGA database, which provides U (unmethylated) and M (methylated) values for each probe. The Beta values of the probes were calculated by M/(U+M+100) [19]. The probe IDs were mapped to Gene IDs with the annotation table for each platform.

**Table 1:** The datasets of nine cancer types for analyzing batch effects.

Cancer type	Abbreviation	Number of batch	Number of Laboratory	Number of Tumour samples	Number of normal samples
Ovarian serous cystadenocarcinoma	OV	13	17	520	35
Colon adenocarcinoma	COAD	9	5	168	23
Lung adenocarcinoma	LUAD	4	11	128	27
Lung squamous cell carcinoma	LUSC	5	12	115	31
Stomach adenocarcinoma	STAD	3	3	82	61
Kidney renal clear cell carcinoma	KIRC	6	10	219	205
Glioblastoma multiforme	GBM	9	13	264	5
Breast invasive carcinoma	BRCA	3	9	186	2
Rectal adenocarcinoma	READ	5	4	70	7

**Table 2:** The Methylation and Expression datasets of five cancer types for concordance analysis.

Cancer type	Methylation#	Database	Expression#	Database
Colon adenocarcinoma	C22	TCGA	c23	GSE4183
	C44	GSE17648	c64	GSE8671
Kidney renal clear cell carcinoma	K78	TCGA	k20	GSE6344
	K100	TCGA	k34	GSE15641
Stomach adenocarcinoma	S24	TCGA	NA	
	S94	TCGA		
Lung adenocarcinoma	La8	TCGA	la52	GSE7670
	La14	TCGA	la107	GSE10072
Lung squamous cell carcinoma	Ls24	TCGA	NA	
	Ls28	TCGA		
Platform	Illumina HumanMethylation27 BeadChip		Affymetrix Human Genome U133 (GPL96,GPL570)	

# Each dataset is denoted by the following nomenclature: initial character of the cancer type followed by the total number of samples of the dataset; NA, not available.

### Analysis of batch effects

The batch effects could be generated for samples from different experimental batches or collection centres in the TCGA database [16]. For the methylation data from TCGA, we computed an F-statistic to test for the correlation between probes' methylation levels (Beta values) and their experimental batches or collection laboratories. The *P* values were adjusted by the Bonferroni-Hochberg procedure with the false discovery rate (FDR)<0.05 [20] and significant probes were considered susceptible to batch effects [9]. To evaluate the effect of experimental batches on DM gene detection, we also compared DM genes selected from datasets compromising tumour samples from different batches and a given group of normal samples from a batch for a particular cancer type.

### Selection of DM genes and DE genes

For each dataset, we selected DM genes with t-test [19] and used the Benjamini-Hochberg procedure to control the FDR at a given level [20]. The DM genes with larger means of methylation levels in the cancer samples than in the normal samples were defined as hypermethylated genes; otherwise, the DM genes were defined as hypomethylated genes.

Differentially expressed (DE) genes were selected using the SAM (significance analysis of microarray) algorithm [21]. Genes with adjusted P values less than 0.05 were defined as differentially expressed (DE) genes.

### Reproducibility analysis of DM genes and DE genes

Then, we evaluated the reproducibility of DM gene detection by analysing the overlap of the lists of DM genes selected from two datasets for each cancer. If k genes are shared by list 1 with length L_1_ and list 2 with length L_2_, then the POG (percentage of overlapping genes) score from list 1 (or list 2) to list 2 (or list 1) is POG_12_ = k/L_1_ (or POG_21_ = k/L_2_). Next, we evaluated the consistency of the methylation directions (hypermethylation or hypomethylation) of the k genes shared by lists 1 and 2 across the two datasets. The same analysis was performed on the lists of DE genes selected from two expression datasets for each cancer.

### Concordance between DNA methylation and gene expression changes

If the expression of a hypermethylated (or hypomethylated) gene was significantly down-regulated (or up-regulated), we considered the methylation change to be concordant to the change in gene expression. We defined the concordance rate between DNA hypermethylation and gene down-regulation as the percentage of down-regulated genes among the hypermethylated genes with differential expression. The *P* value was calculated by the hypergeometric model [10], [11], [12]. Similarly, the concordance rate between DNA hypomethylation and gene up-regulation was defined as the percentage of up-regulated genes among the hypomethylated genes with differential expression.

### Function enrichment of DM genes

Using Elim software, we detected Gene Ontology (GO) terms enriched with DM genes [22]. The *P* values were adjusted by the Bonferroni-Hochberg procedure with an FDR<0.05 [20].

## Results

### Batch effects on DM gene detection

**Figure 1:**
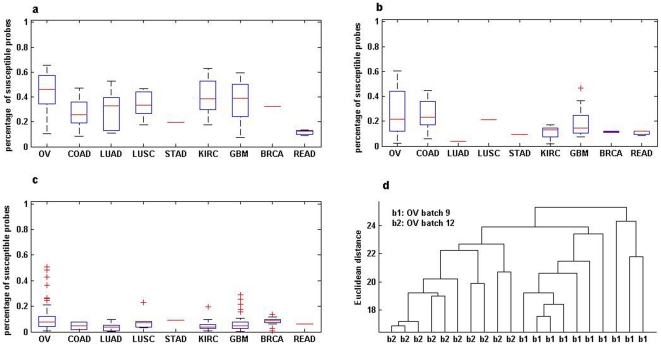
Batch effects on tumour samples for nine cancer types. (a) different batches and different laboratories; (b) the same laboratory but different batches; (c) the same batch but different laboratories; (d) Hierarchical clustering the tumour samples of ovarian serous cystadenocarcinoma in batch 9 and batch 12. For a cancer type denoted in the x-axis in graph a, b or c, a box plot in the y-axis represents the percentage of probes significantly susceptible to different batch conditions. The percentage takes value ranging from 0 (no susceptible probe) to 1 (100% susceptible probes). Each box stretches from the lower hinge (defined as the 25th percentile) to the upper hinge (the 75th percentile) and the median is shown as a line across the box.

We first evaluated the effects of experimental batches on the methylation level for each probe in the tumour samples of two batches separately for nine types of cancers collected in the TCGA database(see Table 1) using the F-statistic with an false discovery rate (FDR)<0.05 [20] (see *Methods*). As shown in Fig. 1a, about 30% of probes, on average, were significantly susceptible to batch effects for the nine cancer types when samples came from different laboratories and different batches. And about 20% probes were still significantly susceptible when restricted samples from the same laboratory but treated in different batches (Fig. 1b). However, only about 7.7% probes were significantly susceptible when samples came from the same batches (Fig. 1c). Especially, as shown in Fig. 1d, the tumour samples from two batches (batch 9 and batch 12) for ovarian serous cystadenocarcinoma could be clustered together perfectly according to batch by the hierarchical clustering algorithm using the Euclidean distances of the Beta values between samples.

**Figure 2:**
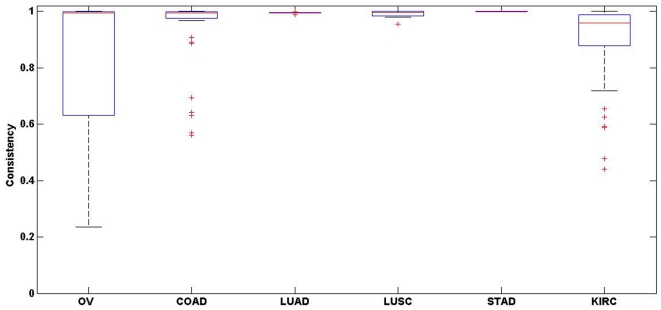
Batch effects on DM genes of six cancer types. For each cancer type denoted in the x-axis, a box plot in the y-axis represents the consistency score defined as the proportion of DM genes with consistent methylation states among all overlapping DM gene commonly detected in both of the two groups (see ‘Methods’ section). The consistency score takes value ranging from 0 (no consistent states) to 1 (100% consistent states). Each box stretches from the lower hinge (defined as the 25th percentile) to the upper hinge (the 75th percentile) and the median is shown as a line across the box.

The above results indicated that integrating tumour samples from different batches to detect DM genes might be misleading. In fact, as a result of the batch effects, the change of methylation states of DM genes in cancer samples compared with normal samples could be highly inconsistent when comparing tumour samples from different batches with the same group of normal controls (see Fig. 2). For example, when comparing tumour samples from batch 9 and batch 15 for ovarian serous cystadenocarcinoma with the same group of normal samples (batch 27), respectively, the consistency of the change of methylation states of the common DM genes was only 23.5%. Therefore, most of the observed differential methylation was across batches rather than across biological groups, leading to highly irreproducible results.

### Reproducibility of DM gene detection

To avoid potential batch effects and bias which could be introduced by different ages of the patients, we only analysed the profiles of five cancer types for each of which pair-matched tumour and normal samples from the same patients collected by the same laboratory and measured in the same experimental batch were available (see Table 2). For each cancer, we used the two largest batches as independent datasets and detected DM genes with t-test at FDR<0.05 [20]. Then, we evaluated the consistency of the two lists of DM genes detected separately from the two datasets (batches) by calculating the percentage of overlapping genes (POG) between the two lists of DM genes [10] (see *Methods*). For each cancer, most of the DM genes on the shorter list were included in the longer list, as reflected by the POG_12_ scores shown in Table 3. More than 99% of the DM genes detected in both datasets were consistent in the change of methylation states across the two datasets. For example, 3778 and 3966 DM genes were separately identified in the two datasets (K78 and K100, respectively) for kidney renal clear cell carcinoma (kidney cancer), with an overlap of 3443 genes. Strikingly, all of the 3443 genes showed the same change of methylation states across the two datasets, significantly more than expected by chance (Bernoulli model *P*<2.2×10^−16^).

**Table 3:** Consistency of DM genes across different datasets for each cancer.

Dataset#	DM-S*	DM-L**	Overlap	POG_12_ $	POG_21_ $$	Consistency¥
C22–C44	2601	4001	2421	93.1%	60.1%	99.9%
K78–K100	3778	3966	3443	91.1%	86.8%	100%
La8–La14	752	1698	488	64.9%	28.7%	99.6%
S24–S94	2274	4867	2210	97.2%	45.4%	100%
Ls24–Ls28	2682	2909	2152	80.2%	74.0%	100%

# Each dataset was denoted by the following nomenclature: initial character of the cancer type followed by the total number of samples of the dataset.

*DM-S denotes DM genes from the shorter list;

**DM-L denotes DM genes from the longer list.

$ POG_12_ denotes the score from the shorter list to the longer list;

$$ POG_21_ denotes the score from the longer list to the shorter list.

¥ Consistency denotes the percentage of overlapping genes which showed the same methylation directions across the two datasets.

A large fraction of DM genes detected in one dataset were not determined to be significant in another dataset for each cancer, as reflected by the POG_21_ scores shown in Table 3. However, our analysis showed that most of the DM genes that were solely detected in one dataset also showed the same change of methylation states in another dataset for the same cancer, revealing that the effective biological signals of these DM genes also existed in the other dataset. For example, for kidney cancer, 514 (98.2%) of the 523 genes detected to be significant solely in the larger dataset (K100) showed the same change of methylation states in the smaller dataset (K78), which was highly unlikely to happen by chance (Bernoulli *P*<2.2×10^−16^). Thus, the relatively low POG_21_ scores might reflect a reduced statistical power for detecting DM genes in the smaller dataset, coupled with a stringent FDR control [10], [23].

We also analysed an independent dataset for colon cancer available from the GEO database [17]. With FDR<0.05, 2601 and 4001 DM genes were identified in the C22 dataset (from TCGA) and the C44 dataset (from GEO), respectively. These two lists of DM genes shared 2421 genes, among which 2419 (99.9%) showed the same change of methylation states across the two datasets (Bernoulli model *P*<2.2×10^−16^). Among the other 1582 genes that were significant in the larger C44 dataset but not in the smaller C22 dataset, 1502 (94.9%) showed the same change of methylation states in the smaller dataset, significantly more than expected by chance (Bernoulli model *P*<2.2×10^−16^). The high consistency of the change of methylation states for the DM genes across different datasets for the same cancer indicated that DM genes in cancer could be reproducibly detected in high-throughput methylation data.

### Reproducibility of DE gene detection

The TCGA data are also problematic for expression data because only one normal sample were measured in expression for each cancer, which makes the comparison between tumour and normal samples unreliable. Therefore, we selected expression data of matched cancer type from GEO database [17]. For nine cancers analysed above, we were able to find two gene expression datasets for three cancers (see Table 2). For each of these three cancers, using SAM [21] with FDR<0.01, we selected two lists of differentially expressed (DE) genes from the two datasets and found that most of the DE genes in the shorter list were included in the longer list, as reflected by the POG_12_ scores shown in Table 4. In addition, over 94.5% of the DE genes detected in both of the datasets for each cancer were consistent in the regulation direction (up or down) across the two datasets, which was highly unlikely to happen by chance (Table 4, Bernoulli model *P*<2.2×10^−16^). In addition, most of the DE genes solely detected in one dataset showed the same regulation directions in another dataset for the same cancer, revealing that the effective biological signals of these DE genes existed in the later dataset. For example, for colon cancer, 6056 (94.5%) of the 6420 genes detected to be significant solely in the larger dataset (c64) showed the same regulation direction in the smaller dataset (c23), which is highly unlikely to happen by chance (Bernoulli model *P*<2.2×10^−16^).

**Table 4:**
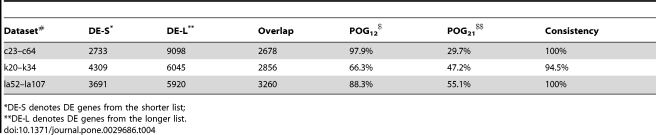
Consistency of DE genes across different datasets for each cancer.

*DE-S denotes DE genes from the shorter list;

**DE-L denotes DE genes from the longer list.

The above analyses were based on data normalised by the RMA algorithm, which assumes that the majority of genes are not differentially expressed in a disease [24]. We performed the same analyses using the least-variant set (LVS) algorithm [25], which relies less on this assumption, and the results were similar.

### Concordance between differential methylation and differential expression

The above results indicated that the methylation and expression changes could be reproducibly detected across different datasets for a particular cancer. Notably, although the expression microarray data from different sources, rather than the TCGA data itself, the highly consistency of expression change across two datasets from the same cancer indicated the gene regulation directions were reproducible and reliable for the specific type of cancer. Therefore, based on the reproducible DM and DE genes of the same cancer type, we examined the influence of gene promoter methylation on gene expression. Briefly, if a hypermethylated (or hypomethylated) gene found by methylation data was significantly down-regulated (or up-regulated) in the expression data, we considered that its DNA methylation was concordant to its expression change. The concordance rate was measured by the percentage of hypermethylated (or hypomethylated) genes concordant to gene down-regulation (or up-regulation).

We evaluated the concordance between differential methylation and expression at two levels. First, we evaluated the concordance between differential methylation and differential expression of genes. As shown in Table 5, 91.6%, 86.6% and 88.2% of the hypermethylated genes were down-regulated in colon, kidney and lung cancers, respectively, indicating that hypermethylation is significantly correlated with down-regulation of genes (hypergeometric test *P*<1.0×10^−5^ for all three cancers). For example, in colon cancer, 98 of the 107 hypermethylated genes were down-regulated in cancer samples compared with normal controls (hypergeometric test *P* = 7.8×10^−9^). Then, we focused on the concordance between methylation with great methylation level change and expression with great fold change (FC) between tumour and normal samples. When we focused on DM genes with at least 0.15 Δβ (difference of the mean methylation levels between tumour and normal samples), the concordance rates increased to 96.1%, 96.2% and 91.3% for colon, kidney and lung cancers, respectively. Similarly, when we focused on reproducible DE genes with at least a 2-fold change (FC), the concordance rates for the three cancers were all above 90%. However, the relationship between the hypomethylation of genes and the up-regulation of gene expression is rather elusive. The concordance rates were 50.3%, 39.4% and 62.5% for colon, kidney and lung cancers, respectively. For lung cancer only, the hypomethylation showed a significant inverse correlation with gene up-regulation (hypergeometric test *P* = 4.2×10^−6^). When we focused on DM genes with at least 0.15 or 0.3 Δβ, the hypomethylation was significantly correlated with the up-regulation of gene expression only in lung cancer. When we examined the DE genes with at least a 2-fold change, the concordance rates increased to 58.5% and 61.7% for colon and kidney cancers, respectively, and became significant (hypergeometric test *P* = 2.7×10^−7^ and 5.4×10^−4^, respectively). Notably, the concordance rates were approximately 60% even after the FC cut-off for the three cancers. These results suggest that hypomethylation may partially affect up-regulation of gene expression with large fold changes.

**Table 5:** Concordance between differential methylation and differential expression.

Cancer types	Hypermethylation			Hypomethylation		
	Gene§ number	Concordance rate	*P* value	Gene§number	Concordance rate	*P* value
Colon	107	91.6%	7.7*10^−9^	157	50.3%	0.99
Kidney	254	86.6%	1.5*10^−12^	302	39.4%	0.397
Lung	34	88.2%	1.5*10^−6^	88	62.5%	4.2*10^−6^

§ Gene number denotes the number of hypermethylated (or hypomethylated) genes which were determined to be differentially expressed in the expression data.

### Functions of hypermethylated genes and hypomethylated genes

Using Elim software with FDR<0.05 [22], we detected GO terms significantly enriched with hypermethylated genes reproducibly identified in the two datasets for each cancer. For colon cancer, we found 58 significant terms, which were associated with basic biological processes such as transcription, cell adhesion and signalling (Supplementary Table S1 for detailed terms). For kidney cancer, we found 14 significant terms, among which 11 were included in the significant terms for colon cancer, suggesting that hypermethylated genes in these two cancers tend to be involved in similar functions. However, no significant GO term was found for lung cancer with FDR<0.05. By comparing the top 10 terms with the smallest *P* values for the three cancers, we found that 4 terms were shared by colon and kidney cancers, and neither cancer shared a term with lung cancer. These results indicated that the hypermethylation pattern of lung cancer may be different from those of colon and kidney cancers.

With FDR<0.05, we found 14, 29 and 2 GO terms enriched with hypomethylated genes for colon, kidney and lung cancers, respectively (Supplemental Table S2). Most of these significant terms were related to immune response. A comparison of the lists of the top 10 terms with the smallest *P* values for the three cancers showed that they shared three terms: ‘keratinization’, ‘defense response to bacterium’, and ‘cellular defense response’. We additionally tested the function of hypomethylated genes from Lung squamous cell carcinoma and Stomach adenocarcinoma data. These genes were also enriched in ‘keratinization’ and ‘defense response to bacterium’ (Supplemental Table S3). Specifically, in‘keratinization’, we found that 12 KAP genes encoding keratin associated proteins (Table 6) were hypomethylated in all five types of cancers. Notably, these 12 KAP genes were also included in the 16 KAP genes found to show pronounced differential hypomethylation in bladder cancer [26]. These evidences together suggest that KAP genes could be used as biomarkers for multiple cancers. Finally, a comparison of two of the three cancers revealed that the DM genes detected solely in a particular cancer were more likely to be hypermethylated than the DM genes detected in two cancers (chi-squared test *P*<0.001 for the comparison of the proportions of hypermethylated genes). For example, 635 (43.5%) of the 1411 DM genes detected in colon cancer but not in lung cancer were hypermethylated, while only 42 (16.5%) of the 254 DM genes detected in both cancers were hypermethylated. On the other hand, 168 of the 189 DM genes shared by the three cancers were hypomethylated and enriched in ‘keratinization’, ‘chemotaxis’, and ‘immune response’ functions (see *Discussion*).

**Table 6:** Keratin associated protein genes hypomethylated in five cancers.

GeneID	Gene Name	GeneID	Gene Name
337880	keratin associated protein 11-1	337972	keratin associated protein 19-5
140258	keratin associated protein 13-1	337976	keratin associated protein 20-2
337960	keratin associated protein 13-3	337977	keratin associated protein 21-1
284827	keratin associated protein 13-4	337978	keratin associated protein 21-2
254950	keratin associated protein 15-1	337979	keratin associated protein 22-1
337882	keratin associated protein 19-1	337879	keratin associated protein 8-1

## Discussion

The detection of aberrant DNA methylation in cancer can yield important biomarkers for predicting cancer outcomes and drug targets. However, pitfalls in experiment designs and faulty data analyses, such as improperly integrating batches of TCGA data, may produce unreliable biomarkers [9]. Notably, most studies using the TCGA data, including many published in high-profile journals [16], [27], [28], [29], did not considered the potential batch effects, which would be likely to produce misleading results associated with the batches rather than the biological outcomes. For example, Houtan et al. [27] integrated glioblastoma tumour samples from several batches and identified a distinct subset of samples displaying concerted hypermethylation, which might have been correlated with their experimental batches similarly to the data shown in the clustering map in Fig. 1d. Therefore, we suggested that the conclusions based on integrated samples should be re-evaluated by considering potential batch effects. Our results strongly suggest that, an experiment should be designed to avoid the batch effect by equally distributing possible experimental surrogates between biological groups and using sufficient samples for each group [9].

Our results showed that DM genes detected from different datasets for the same cancer, excluding batch effects, were consistent in methylation across the datasets, similar to the observation that DE genes detected from various microarray studies show a consistent up or down expression pattern [10], [30], [31]. Thus, the signals of the methylation states of DM genes in cancer can be reliably detected in methylation arrays. Notably, 36 of the 47 hypermethylation genes of colon cancer documented in the Methycancer database [32] were found to be DM genes in our colon cancer data, among which 34 were also hypermethylated (Supplementary Table S4). The reproducible methylation biomarkers in different cohorts of patients could provide valuable information for finding prognostic biomarkers and drug targets for cancers.

On the other hand, we found that, for a particular cancer, many DM genes detected in one dataset may not be significant in another dataset due to the insufficient power of detecting DM genes in small samples coupled with stringent FDR control [10], [30], [33]. The reduction of power could lead to the selection of the most significant genes as biomarkers for a cancer to be highly unstable across different studies [34]. To evaluate the reproducibility of the most significant DM genes discovered from different studies for a particular cancer, we could take into account the functional relationship rather than simply counting the overlaps [11], [35].

For the function of DM genes, our results showed hypermethylation of gene promoters was significantly linked to the down-regulation of gene expression in cancer and affects basic biological processes, such as signalling and cell growth, similar to what has been observed for human ageing [36]. By contrast, hypomethylation was only weakly correlated with gene up-regulation, indicating that other factors such as gene body hypermethylation [37] and copy amplification [38] may contribute more to the up-regulation of gene expression. We found that hypomethylated genes for different cancers were similar in functions directly linked to chronic inflammation, such as ‘chemotaxis’ and ‘immune response’. Chemokines play important roles in regulating inflammation progress [39], and immune deficiency can result in chronic inflammation [39]. This chronic inflammation may induce global hypomethylation, which may cause chromosome instability and increase mutations of the genome and then increase the risk of cancer [40].

Additionally, our results showed that DM genes detected in a specific type of cancer were more likely to be hypermethylated than DM genes detected in multiple cancers. However, defining cancer type-specific biomarkers is difficult because different studies for a particular cancer frequently discover different DM genes. Using the tissue-specific genes collected by Xiong et al. [41], we found that genes preferentially expressed in a specific tissue were enriched with genes differentially methylated in the corresponding cancer type (hypergeometric test *P*<0.001 for all three cancers), but these DM genes did not show any preference toward hypermethylation or hypomethylation. Considering that the accuracy of “tissue-specific” genes strongly depends on the expression level of the respective transcript [42], it might be more reliable to define “tissue-specific” genes by their methylation patterns [43]. In future work, we plan to study cancer type-specific DM genes by taking into account the opposite methylation directions of DM genes detected for different cancer types.

## Supporting Information

Table S1 GO terms significantly enriched with Hypermethylation genes separately for Colon, Kidney and Lung cancers.
(XLS)

Table S2 GO terms significantly enriched with Hypomethylation genes separately for Colon, Kidney and Lung cancers.
(XLS)

Table S3 GO terms significantly enriched with Hypomethylation genes separately for Lung squamous cell carcinoma and Stomach adenocarcinoma.
(XLS)

Table S4 Methylation and expression changes of genes in the MethyCancer database.
(XLS)
